# Fine-grained recognition of bitter gourd maturity based on Improved YOLOv5-seg model

**DOI:** 10.1038/s41598-024-61635-w

**Published:** 2024-05-13

**Authors:** Sheng Jiang, Jiangbo Ao, Hualin Yang, Fangnan Xie, Ziyi Liu, Shanglin Yang, Yichen Wei, Xijin Deng

**Affiliations:** 1 College of Electronic Engineering, South China Agricultural University, Guangzhou, 510642 China; 2 Qinghai Provincial Highland Rural Informatization Engineering Technology Research Center, Xining, China; 3 Guangzhou Hoire Intelligent Technology Co., Ltd, Guangzhou, China

**Keywords:** Electrical and electronic engineering, Mechanical engineering, Computer science

## Abstract

Bitter gourd, being perishable, requires timely harvesting. Delayed harvesting can result in a substantial reduction in fruit quality. while premature harvesting leads to underdeveloped fruit and decreased yields, the continuous flowering pattern in bitter gourd underscores the significance of accurately assessing fruit growth and ensuring timely harvesting for subsequent fruit setting and development. The current reliance on the experience of production personnel represents a substantial inefficiency. We present an improved real-time instance segmentation model based on YOLOv5-seg. The utilization of dynamic snake convolution enables the extraction of morphological features from the curved and elongated structure of bitter gourd. Diverse branch blocks enhance feature space diversity without inflating model size and inference time, contributing to improved recognition of expansion stages during bitter gourd growth. Additionally, the introduction of Focal-EIOU loss accurately locates the boundary box and mask, addressing sample imbalances in the L2 stage. Experimental results showcase remarkable accuracy rates of 99.3%, 93.8%, and 98.3% for L1, L2, and L3 stages using mAP@0.5. In comparison, our model outperforms other case segmentation models, excelling in both detection accuracy and inference speed. The improved YOLOv5-seg model demonstrates strong performance in fine-grained recognition of bitter gourd during the expansion stage. It efficiently segments bitter gourd in real-time under varying lighting and occlusion conditions, providing crucial maturity information. This model offers reliable insights for agricultural workers, facilitating precise harvesting decisions.

## Introduction

Bitter gourd (*Momordica* *charantia* L.) stands out as a distinctive and versatile melon, garnering significant popularity in Asia. It has a long, thin curved shape and green skin. Belonging to the gourd family, bitter gourd boasts a unique bitter taste, enriched nutritional value, and a distinctive appearance, endearing it to a vast consumer. Its medicinal properties, such as promoting skin health, aiding in weight loss, and providing anti-diabetic benefits, have earned it the moniker “plant insulin”. In orchards, the conventional method of manually picking bitter gourd relies largely on accumulated experience, where attributes like large volume and a glossy surface are deemed suitable indicators. However, the picking criteria for bitter gourd often remain ambiguous. Research indicates that^1^ the optimal picking time is 10–15 days after blooming, yet the asynchronous flowering period on bitter gourd vines results in fruits of varying maturity on the same plant. This lack of uniformity hinders efficient management and picking. To address these challenges, there is a pressing need for an accurate and real-time method to estimate the maturity of bitter gourd. This development is essential to align with storage and transportation requirements, enhance yield and quality, and overcome the difficulties and inefficiencies associated with manual picking. An immediate and precise solution is required to assist farmers in the effective management of bitter gourd cultivation.

Advancements in computer vision applications for fruit maturity estimation within the agricultural sector have demonstrated significant progress. Traditional machine learning methods are commonly employed to distinguish various stages of fruits, primarily relying on color features. This approach is particularly evident in the classification of fruits such as grapes^2^, strawberry^3^, banana^4^, and other fruits. Tan et al.^5^ proposed a distribution algorithm for discerning the maturity of blueberry fruits. The algorithm utilized a Histogram of Oriented Gradients (HOG) feature vector for training a Support Vector Machine (SVM) classifier to swiftly identify regions of fruit style. K-Nearest Neighbors classifiers were employed to differentiate fruits at different maturity levels. The prevalence of deep learning has precipitated a surge in research on fruit ripeness recognition. A comprehensive survey by Matteo Rizzo et al.^6^ Delving into fruit ripeness classification, the survey concluded that pre-processing and fine-tuning of deep learning models is the most promising approach. Faisal et al.^7^ introduces a multi-stage intelligent harvest decision system for date palm recognition, employing pre-trained models like VGG-19^8^, Inception-V3^9^, and NASNet^10^ architectures. In another study, Chen et al.^11^ proposed an improved EfficientDet^12^ method for olive fruit maturity estimation, which introduced Convolution Block Attention Module (CBAM)^13^ into the feature extraction network to refine the feature mapping between different rims. The precision, recall and mAP of the improved EfficientDet model in the test set were 92.89%, 93.59% and 94.60%, respectively, by enhancing information flow in the feature pyramid network. The most advanced techniques in maturity estimation include the two-stage Region-based Convolutional Neural Network (RCNN)^14–16^ and the one-stage You Only Look Once (YOLO)^17–19^ series. Tu et al.^20^ introduced a two-stage Faster R-CNN model for passion fruit estimation, employing dense scale-invariant feature transform and local constrained linear coding for feature extraction, followed by SVM classification. The continuous evolution of deep learning has significantly enhanced detection speed and accuracy. Tian et al.^21^ proposed an improved YOLOv3 for detecting apples at various growth stages, demonstrating robust performance in scenarios with occlusion and overlapping conditions. Wei et al.^22^ introduce the Shine-Muscat Grape Detection Model (S-MGDM), a novel approach leveraging an enhanced version of YOLOv3 tailored for detecting the ripening stage of grapes. Central to the methodology is the integration of DenseNet^23^ into the backbone feature extraction network, facilitating the extraction of more comprehensive grape-specific information. Additionally, the multi-scale detection module is augmented with depth-separable convolution, CBAM, and SPPNet^24^ techniques, effectively widening the perceptual field of grape targets while mitigating computational overhead. The S-MGDM model achieves impressive results, boasting an average accuracy of 96.73% on the Sunshine Rose grape test set, with an F1 score of 91%. Similarly, Wang et al.^25^ employed an enhanced YOLOv4-Tiny model to identify blueberries, incorporating an attention module in the object detection network and adopting CSPDarknet53-Tiny as the backbone network. The resulting accuracy of 97.30% in the verification set met the requisite standards for blueberry fruit recognition. notably, Hasan et al.^26^ achieved a recognition rate of 99% using convolutional neural networks to distinguish the growth stages of bitter gourd. but the study only categorized the fruit growth stages of bitter gourd into immature and overripe phases. For farmers, identifying the immature stage does not meet picking standards, and recognizing the overripe stage holds no significance.

However, the above deep learning model can only detect different growth stages of fruit by obvious color features and texture features, while bitter gourd at different stages of expansion can only be judged by the swelling degree of fruit tumor, gully depth, and bumpy phenotype. For fruits with obvious color and texture changes in subcircle, box positioning is sufficient for detection and identification. As for the narrow and long fruit, the horizontal positioning accuracy is insufficient and the identification error is large. Therefore, this study proposes an improved YOLOv5-seg model. The main contributions are as follows: (1) For the slender strip of bitter gourd dataset, we propose to use dynamic snake convolution to fit and segment the object structure, so that the YOLOv5-seg model can better handle the strip continuity structure and pay attention to the core features. (2) On the premise of not increasing model size and inference time, Diverse Branch Block (DBB) is used to enhance the diversity of feature space and strengthen the backbone network feature extraction capability of YOLOv5-seg model. (3) Focal-EIOU loss was introduced to solve the sample imbalance in the L2 phase of bitter gourd dataset and accurately locate the boundary box and mask.

The rest of this study is organized as follows: In the “Methods” section, we introduce the collected dataset of bitter gourd, explain how to correctly label the maturity information during the expansion period, present the improved model of YOLOv5-seg and the configuration and hyperparameters used in experimental training. In the “Results” section, we provide a detailed description of the model's evaluation performance, the achieved results, the interpretation of ablation experimental detailed data, and a comparison of advantages and disadvantages with other mainstream models. The “Discussion” section explores the experiment's key role in smart agriculture, includes existing limitations, and concludes with a summary in the last section.

## Methods

### Data acquisition and processing

**Figure 1:**
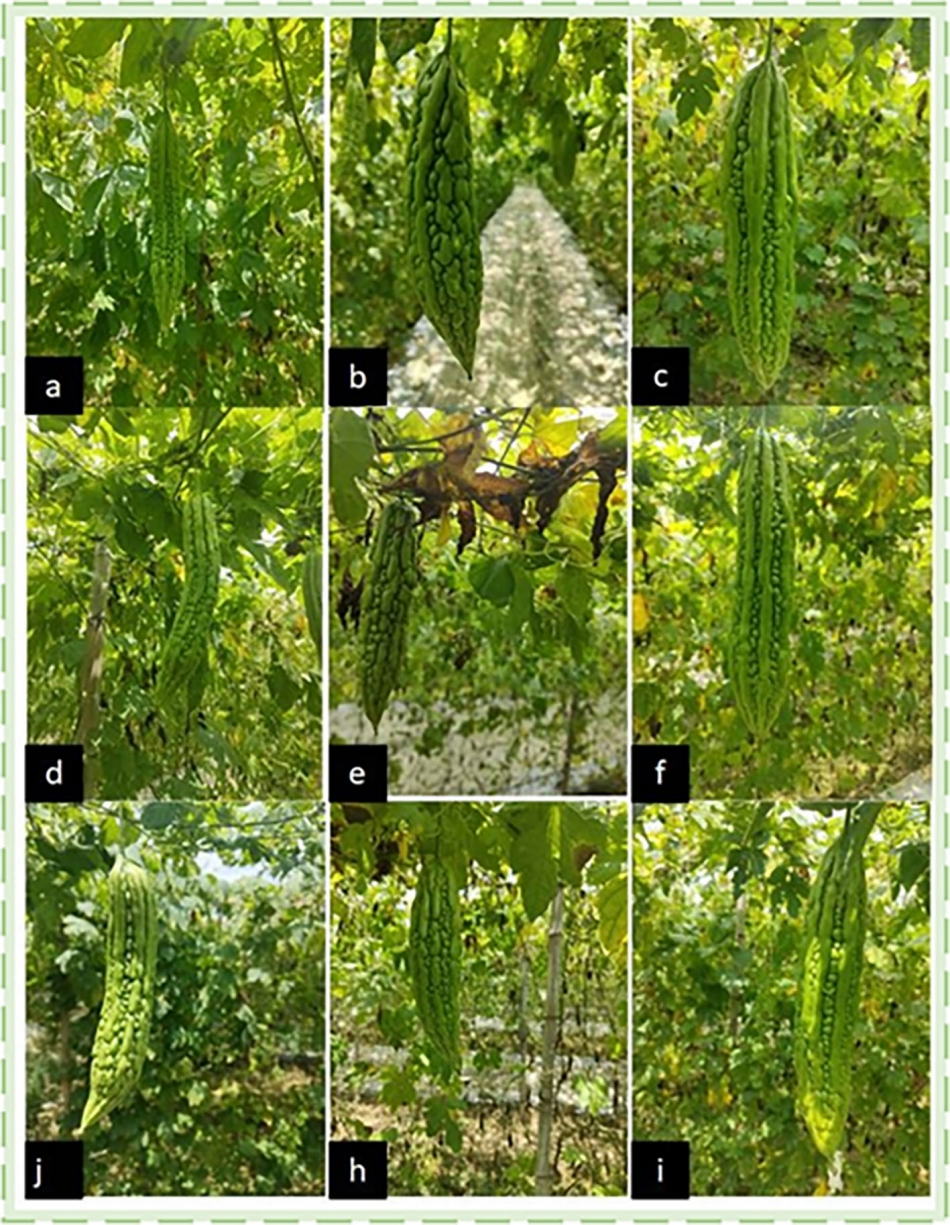
(**a**,**d**,**j**) is the early expansion stage (L1). (**b**,**e**,**h**) is middle expansion stage (L2). (**c**,**f**,**i**) is the late expansion stage (L3).

Seed coat color^1^, pulp color, fruit placenta color and seed weight of bitter gourd in different growth stages are all used as criteria to estimate the maturity of bitter gourd. With the increase of maturity of bitter gourd, the seed coat color will change from cream color or light green brown to pink, but these are from the inside of the fruit, and the external characteristics of the fruit in different periods are the key to explore. Drawing from the insights of experienced pickers and horticultural experts, bitter gourd growth is generally categorized into three stages^27^: the fruity stage, expansion stage and ripening stage. The fruity stage marks the transition from female flower opening to young fruit. The expansion stage signifies rapid fruit expansion before stabilization, and the ripening stage represents the yellowing and softening of the fruit. Of particular interest is the expansion stage, Farmers’ harvesting standards were also set during this period. However, the horticultural definition of the expansion stage is broad and challenging to precisely measure. Through constant observation, the external characteristics of bitter gourd in the expansion stage have obvious characteristics. Early in this stage, the fruit develops into elongated strips with small tumors, and the gullies between these tumors are shallow and uneven. In the middle stage, the fruit tumors rapidly expand, and the gullies deepen, yet there is no distinct smooth straight convex area. In the later stage, the fruit tumors exhibit clear particles and distinct smooth, straight areas. Consequently, the expansion stage of bitter gourd is accurately subdivided into three stages: L1 in the early expansion stage, L2 in the middle expansion stage, and L3 in the late expansion stage, as depicted in Fig. 1. This refined categorization provides a more nuanced understanding of bitter gourd maturity, facilitating precise measurement and informed harvesting decisions during the critical expansion stage.

**Figure 2:**
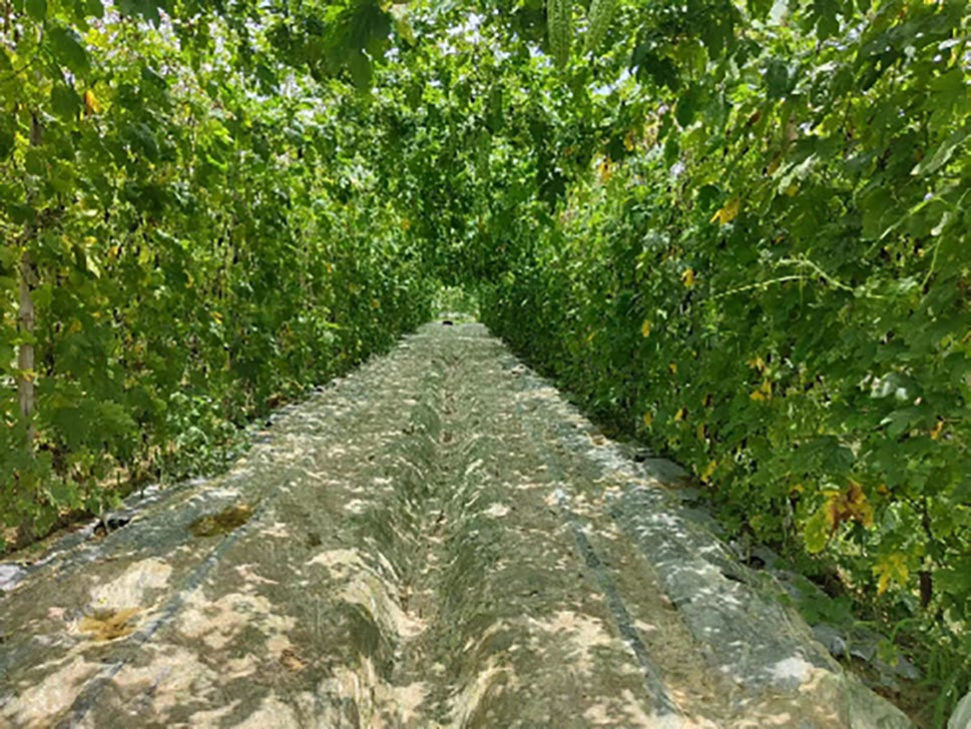
Shooting scene diagram of bitter gourd dataset.

The dataset utilized in this study was captured entirely under natural outdoor lighting conditions. The shooting location was situated within the greenhouse of bitter gourd planting base in Sanjiang Town, Zengcheng District, Guangzhou, Guangdong Province, China, as depicted in Fig. 2. The images were collected under the conditions of clear weather, sufficient light and high definition, under which a total of four times were collected. The initial data collection commenced at noon on May 26, 2023, and concluded on June 20, 2023. A 50-million-pixel camera mobile phone was employed for image capture. To minimize background interference, special attention was given to capturing the fine-grained texture characteristics of bitter gourd during the expansion stage. The shooting distance was carefully controlled, maintaining it within about 1.5 m from the bitter gourd. Additionally, a subset of images was intentionally taken under challenging conditions, including instances of melon overlapping, leaves occlusion, and scenarios where the targets were densely packed with melon. These variations in image conditions aimed to enhance the robustness and real-world applicability of the model developed in this research.

**Figure 3:**
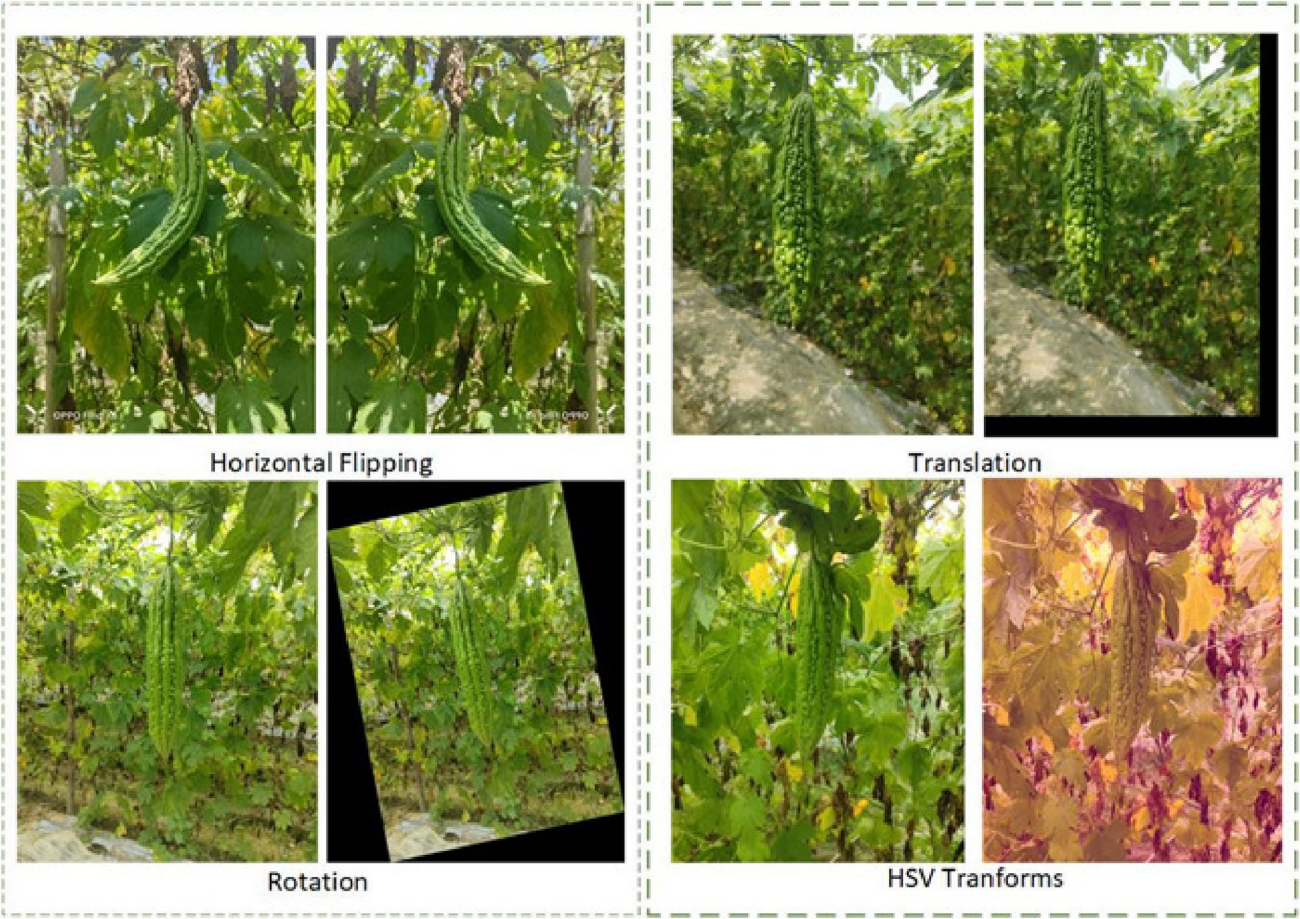
Four methods of image enhancement.

Following the collection phase, the dataset of bitter gourd underwent manual cleaning, resulting in a total of 1121 images. The Labelme annotation software was employed. This tool facilitated the marking of the polygonal areas corresponding to bitter gourd in each image, with the annotations saved in txt format. Comprehensive analysis was conducted on all 1121 images, and the results were compiled to generate bitter gourd instance objects. Subsequently, in order to further expand the enhanced bitter gourd dataset and enable the model to have strong generalization ability on complex scenes, we adopted data augmentation methods, as shown in Fig. 3, including Horizontal Flipping, Translation, Rotation and HSV transforms. As a result, we obtained a total of 2242 final images of bitter gourd dataset, the dataset is divided into training set, verification set and test set according to 7:2:1. The detailed division of bitter gourd dataset is shown in Table 1.

**Table 1:** Bitter gourd dataset partition.

Bitter gourd dataset	L1	L2	L3
Training set (1568 images)	774	464	368
Validation set (450 images)	244	134	108
Test set (224 images)	116	70	62

### Bitter gourd instance segmentation

**Figure 4:**
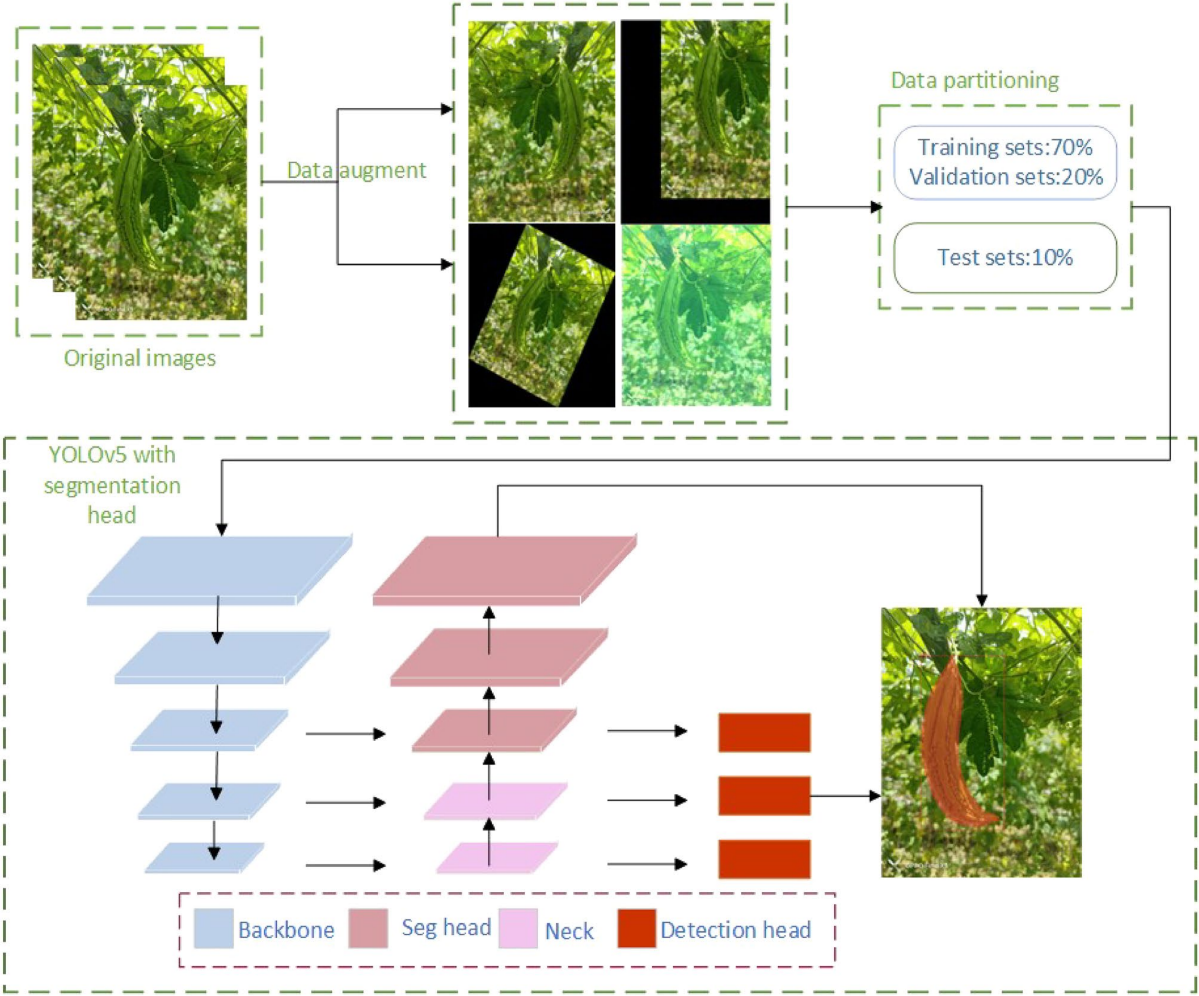
The workflow of improved YOLOv5-seg.

In the context of bitter gourd instance segmentation, compared to box positioning detection, instance segmentation provides precise positional data for automated picking robots. It accurately defines object boundaries and shapes, individually analyzing and annotating each instance of bitter gourd within the image, thereby offering a detailed understanding of the characteristic information associated with different instances of bitter gourd. This multi-task learning model incorporates variations and correlations among different tasks, thereby enhancing the model's generalization capabilities. Notably robust against real farm background interference, instance segmentation also provides semantic insights into the object, specifying its exact location within the surrounding environment, thus demonstrating commendable performance across various practical application scenarios. In 2022, the Ultralytics team introduced YOLOv5-seg, an innovative instance segmentation model. Drawing inspiration from the YOLACT^28^ instance segmentation model and the detection capabilities of YOLOv5, it adopts a lightweight network architecture and efficient inference algorithms. This design optimization significantly improves detection speed without compromising accuracy. Our Improved YOLOv5-seg model workflow is shown in Fig. 4, followed by a detailed explanation of the experimental process and methods.

### Improved YOLOv5-seg model

#### Dynamic snake convolution

**Figure 5:**
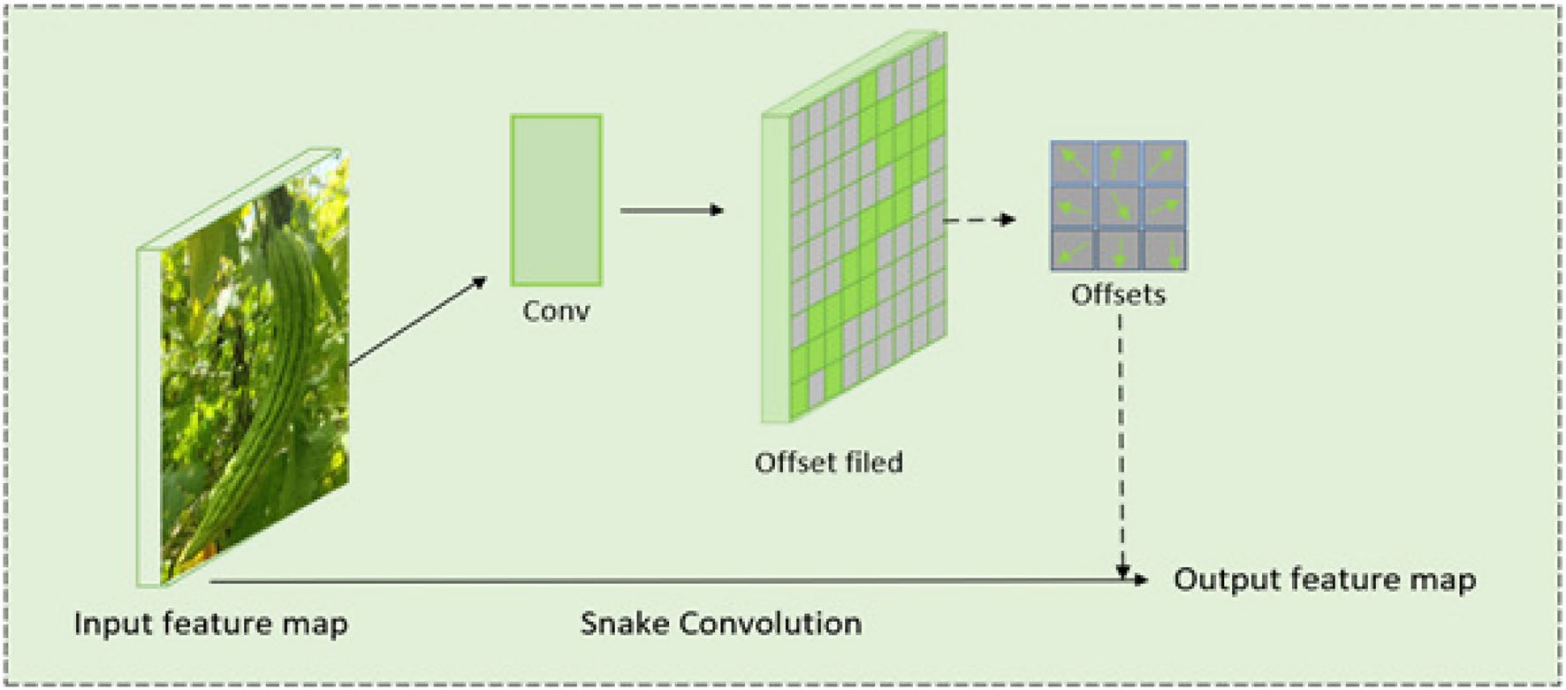
Dynamic Snake Convolution (DS Conv).

Dynamic snake convolution (DS Conv) is a deformable convolution structure introduced by Qi^29^. Drawing inspiration from the crawling motion of snakes, this convolutional method excels in extracting local features of tubular structures. Its adaptability allows it to selectively focus on elongated and tortuous local structures, ensuring the accurate capture of features specific to tubular shapes. To prevent the local receptive field of the model from deviating from the target, a situation that could lead to the loss of surrounding target features during prolonged training, an iterative strategy was introduced. This strategy prevents scattered jumping, maintaining continuity in the receptive field. The DS Conv structure is illustrated in Fig. 5. The DS Conv process comprises two main components: (1) Offset Calculation: A portion of the offset field in both the X and Y directions is derived through the convolution of the input feature map. This offset field, serves to dynamically adjust the shape and position of convolutional nuclei in each application. In contrast to common convolutional approaches with square regions, the use of offsets allows for dynamic adaptation, optimizing the shape and position of the convolutional kernel. (2) Output Feature Map: The input feature map undergoes convolution through the convolution kernel of offsets to generate the output feature map. This unique convolution kernel facilitates easier learning of segmentation, fitting bar structures more effectively, and prioritizing core features.

Conceivably, some of the key technologies in image processing applications are also inspired by biological behaviors, such as ant colony^30^ foraging, echolocation by bats^31^ and dolphins^32^. The foundation of these similar forms of motion in image processing comes from the chain coding, proposed by Freeman^33^ in 1961. Chain coding is a method for representing image contours, converting continuous pixel contours in an image into a series of connected chain codes. Chain coding encodes the shape information of the contour and the connection relationship between the points on the contour into a sequence, making it convenient to represent and process the image contour. Building upon the movement behavior observed in ant colony foraging, Mouring et al.^30^ proposed a new image coding method inspired by ant movement trajectory, which offers a higher compression ratio and is easier to implement compared with other methods. Other recent trends in research have also drawn inspiration from biological behaviors for chain coding movements, addressing many real-world problems and exploring new aspects from various angles.

#### Diverse branch block module

The diverse branch block (DBB) is a versatile building block for convolutional neural networks, introduced by Ding et al.^34^ with the goal of enhancing the model's feature extraction capability and robustness. In a previous study, Zhang et al.^35^ successfully incorporated the DBB module into the YOLOX-S model, demonstrating improved feature extraction capabilities and achieving strong performance on open datasets. The DBB module achieves this by enriching the feature space through the combination of different branches with varying scales and complexities, thereby enhancing the representation capability of individual convolutions. Similar to the Inception architecture, the inclusion of various receptive fields and multi-path convolution operations with different complexities serves to improve feature extraction capabilities. the DBB includes six types of transformations: batch normalization (BN), branch addition, deep concatenation, multi-scale operation, average pooling, and convolution sequence. $I \in {R^{C \times H \times W}}$ is the input, $O \in {R^{D \times H^{\prime} \times W^{\prime}}}$ is the output, $F \in {R^{D \times C \times K \times K}}$ is the K × K convolution kernel, and b is the offset term of the K × K convolution kernel. The convolution operation formula can be expressed as follows:

1 $$O = I \otimes F + REP(b)$$

here $\otimes$ represents the convolution operation and ${\text{REP}}({\text{b}}) \in {{\text{R}}^{D \times H^{\prime} \times W^{\prime}}}$ represents the bias after the operation. Usually, a BN layer is added after the unbiased term, and the value on the jth output channel is determined by the following Eq. (2):

2 $${O_{j,:,:}} = \left( {{{\left( {I \otimes F} \right)}_{j,:,:}} - {\mu_j}} \right)\frac{{\gamma_j}}{{\sigma_j}} + {\beta_j}$$

where ${\mu_j}$ and ${\sigma_j}$ are the mean value and standard deviation of BN, ${\gamma_j}$ and ${\beta_j}$ are the scale factors and deviation terms of learning. Using $F_{j,:,:,:}^{\prime}$ to replace $\frac{{\gamma_j}}{{\sigma_j}}{F_{j,:,:}}$ and $b_j^{\prime}$ to replace $- \frac{{{\mu_j}{\gamma_j}}}{{\sigma_j}} + {\beta_j}$, a BN fusion formula is obtained, then Transform I:

3 $$O_{j,:,:} = I \otimes F_{j,:,:,:}^{\prime} + b_j^{\prime}.$$

Since two or more convolution branches with the same configuration are additive. Additivity can combine the outputs of multiple convolution with the same configuration into a single convolution. The branch merging of Transform II is as follows:

4 $$F^{\prime} \leftarrow {F_1} + {F_2},\;\;b^{\prime} \leftarrow {b_1} + {b_2}$$

Transform III is to merge a sequence of 1 × 1 conv-BN-K × K conv-BN into one single K × K conv. Perform transformation I to obtain Conv 1 × 1 Conv-K × K Conv. With ${F_1} \in {R^{D \times C \times 1 \times 1}}$ as the convolution kernel of 1 × 1 Conv and ${F_2} \in {R^{E \times D \times K \times K}}$ as the convolution kernel of K × K Conv. Their biases are ${b_1} \in {R^D}$ and ${b_2} \in {R^E}$. The output is

5 $$O^{\prime} = \left( {I \otimes {F_1} + REP\left( {b_1} \right)} \right) \otimes {F_2} + REP\left( {b_2} \right).$$

The Eq. (5) for fusion is as follows:

6 $$O^{\prime} = I \otimes F^{\prime} + REP\left( {b^{\prime}} \right)$$

According to the additivity of the branch merging and convolution above, we can get:

7 $$O^{\prime} = I \otimes {F_1} \otimes {F_2} + REP\left( {b_1} \right) \otimes {F_2} + REP\left( {b_2} \right).$$

since $I \otimes {F_1}$ performs a 1 × 1 linear transformation, such a transformation can be achieved by transposing convolution, ditto, the transposed term is transformed as follows:

8 $$F^{\prime} = {F_2} \otimes TRANS\left( {F_1} \right)$$

9 $$REP\left( {b^{\prime}} \right) = REP\left( {b_1} \right) \otimes {F_2} + REP\left( {b_2} \right) = REP\left( {\hat b} \right) + REP\left( {b_2} \right)$$

Transform V is the average pooling layer changes the K × K convolution into a volume with a certain step length, and the average pooling convolution with a convolution kernel of K and a step length of S is equivalent to replace the C-channel feature map with $F^{\prime} \in {R^{C \times C \times K \times K}}$, which is composed of the following Eq. (10):

10 $$F_{d,c,:,:}^{\prime} = \left\{ {\begin{array}{*{20}{l}} {\frac{1}{{K^2}}}&{\quad if\;\;d = c,} \\ 0&{\quad elsewise.} \end{array}} \right.$$

The multi-scale convolution fusion of the final Transform VI is to convert ${k_h} \times {k_w}$ $\left( {{k_h} \leqslant K,\;{k_w} \leqslant K} \right)$ convolution kernel to K × K convolution kernel by zero padding.

**Figure 6:**
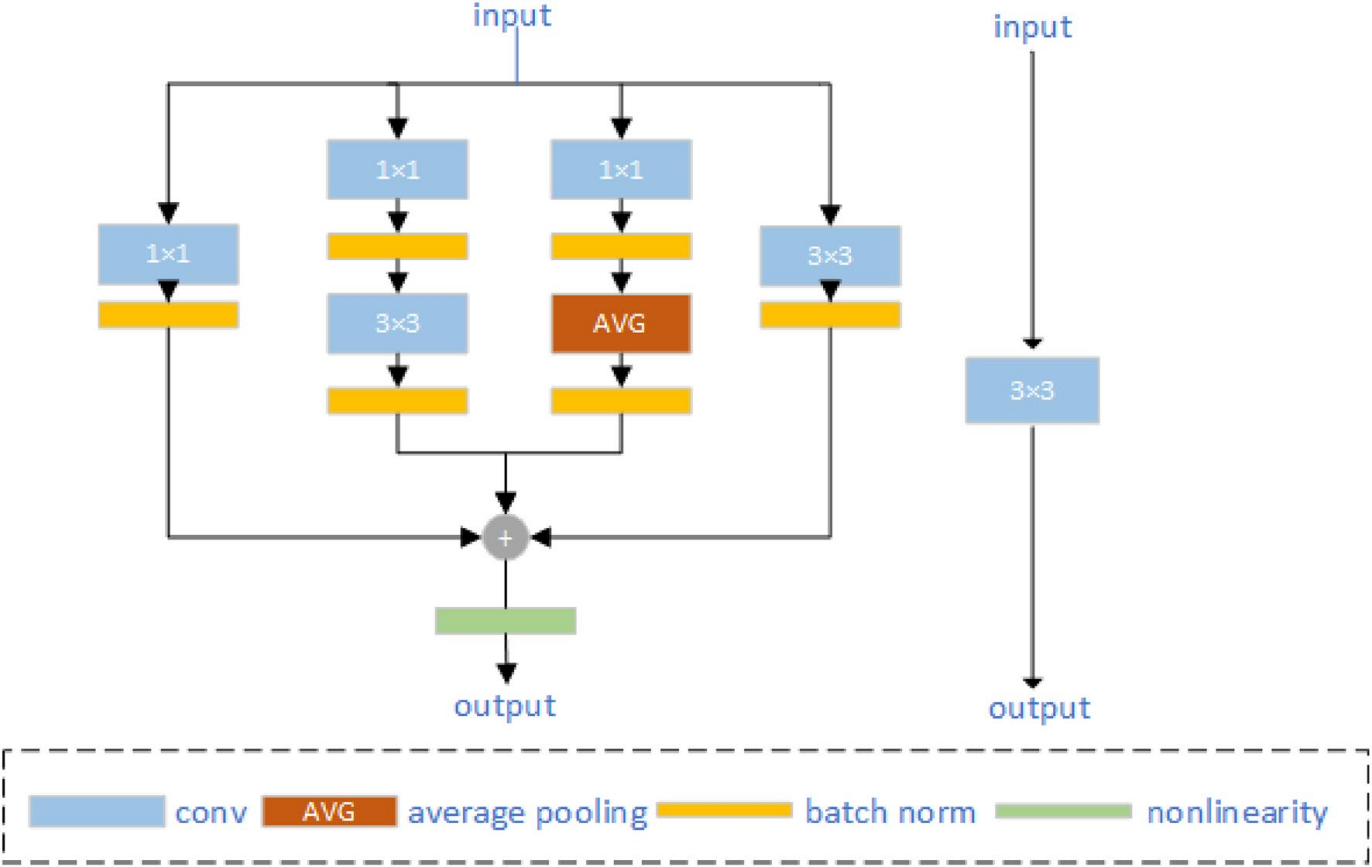
DBB module.

A representative example of the DBB module is shown in Fig. 6. It does not involve the series of the deep series Transform IV^25^. Based on the idea of lightweight network model and heavy parametric structure^36^, a series of combination methods are used to enhance the original 3 × 3 convolution. The 1 × 1 convolution kernel is initialized to the identity matrix, and the other convolution kernels are initialized by default. Each operation has a different sensitivity field and complexity, which can improve the fine-grained recognition ability and greatly enrich the feature space. Finally, a nonlinear layer is added after the convolution operation to improve the nonlinear fitting ability.

#### Focal-EIOU loss

The intersection over union (IOU)^37^ is a metric commonly used to evaluate the performance of target detection models, particularly in the context of two-dimensional collections of rectangular boxes. When a model generates a series of bounding boxes, the IOU is employed to quantify the overlap between the generated bounding boxes and the actual target bounding boxes. The IOU is calculated using the following Eqs. (11) and (12):

11 $$IoU = \frac{A \cap B}{{A \cup B}}$$

12 $${L_{loU}} = 1 - IoU$$

This formula can reflect the difference between positive and negative samples and has scale invariance. However, when two overlapping boxes do not intersect, IOU = 0 and Loss = 0, which cannot reflect the distance gap between boxes and the gradient return update gradient. The YOLOv5 target detection model uses CIOU^38^ as the boundary box loss function, and the calculation Eq. (13) as follows:

13 $$CIoU = IoU - \left( {\frac{{{\rho^2}\left( {b,{b^{gl}}} \right)}}{{c^2}} + \alpha \upsilon } \right)$$

14 $$\upsilon = \frac{4}{{\pi^2}}{\left( {arctan\frac{{{w^{gl}}}}{{{h^{gl}}}} - arctan\frac{w}{h}} \right)^2}$$

15 $$\alpha = \frac{\upsilon }{{\left( {1 - IoU} \right) + \upsilon }}$$

takes into account aspect ratio, α is the weight parameter, υ is used to measure the similarity of aspect ratio and reflect the difference of aspect ratio. The EIOU^39^ Eq. (16) is as follows:

16 $$EIoU = IoU - \frac{{{\rho^2}\left( {b,{b^{g^{\prime}}}} \right)}}{{c^2}} - \frac{{{\rho^2}\left( {w,{w^{g^{\prime}}}} \right)}}{{{c_w}^2}} - \frac{{{\rho^2}\left( {h,{h^{g^{\prime}}}} \right)}}{{{c_h}^2}}$$

17 $${L_{EIoU}} = 1 - EIoU$$

Using the true width and height of the prediction box rather than the aspect ratio for regression, compared with CIOU, eliminates the negative impact of the aspect ratio uncertainty and is more conducive to network performance optimization. Moreover, within the training phase of bounding box regression, the bitter gourd dataset presents a notable challenge due to an excessive number of instance objects in the L1 stage. This results in a sample imbalance issue, with bounding box regression playing a pivotal role in determining target positioning performance. Consequently, to address this concern, the experiment employs Focal-EIOU loss as the loss function for bounding box regression. This choice aims to alleviate the model's tendency to overly concentrate on the expansion stage of L1, thereby offering a more effective measurement of the bounding box's positioning problem. Its formula is:

18 $${L_{Focal - EloU}} = Io{U^\gamma }{L_{EloU}}$$

where γ is a parameter that controls the degree of outlier suppression.

#### Our model

**Figure 7:**
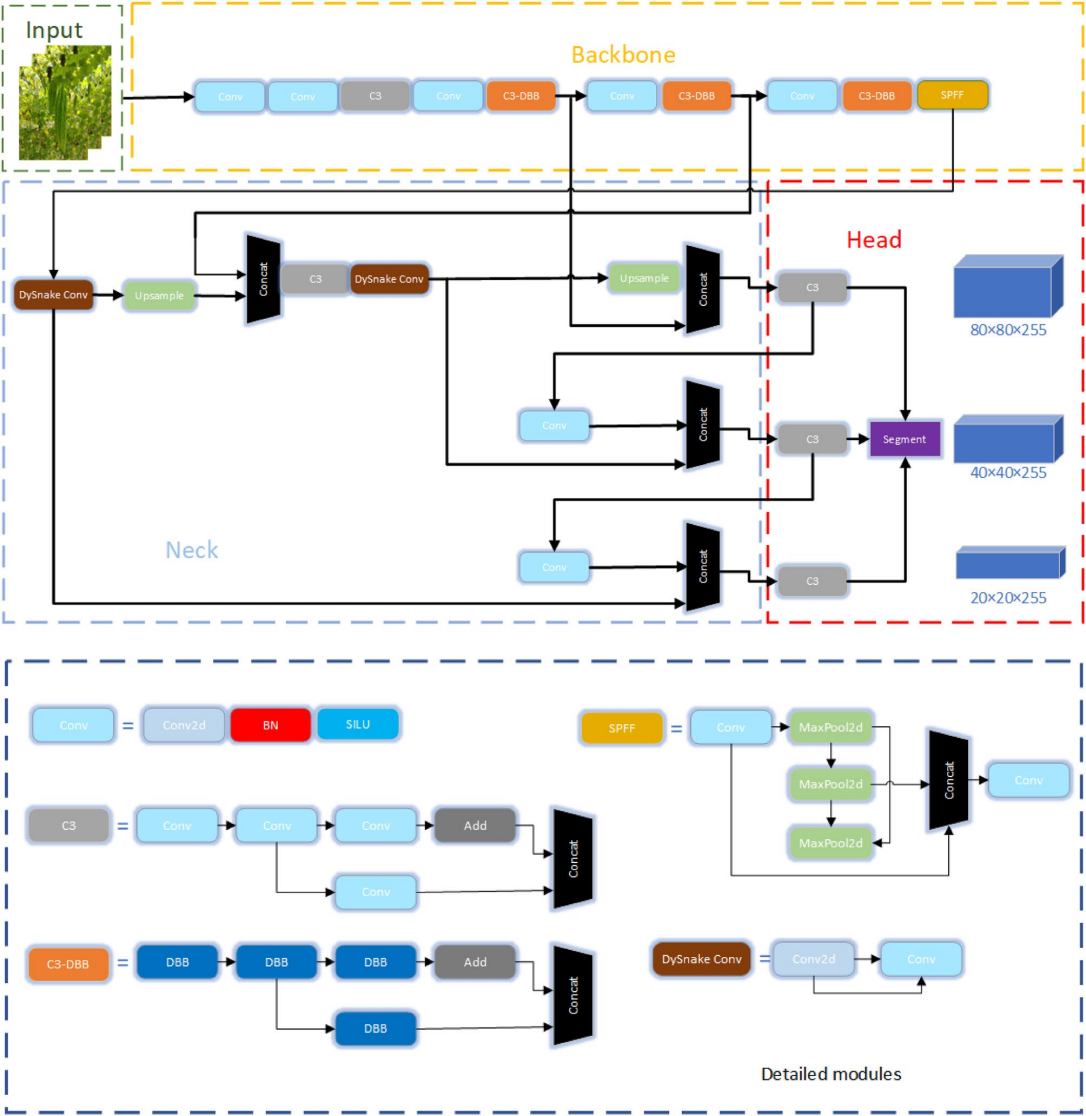
Improved YOLOv5-seg model.

YOLOv5 (You Only Look Once version 5) is widely acclaimed in the industry as a classic model for object detection, exerting a significant influence on computer vision and deep learning. Its key strength lies in processing images through a single-stage convolutional neural network to directly obtain image categories and coordinates. This end-to-end design grants YOLOv5 exceptional real-time performance in object detection, swiftly identifying objects in images or videos. YOLOv5 can detect multiple targets of different categories, providing precise boundary box position information while maintaining real-time performance. The YOLO series has evolved from YOLOv1 to YOLOv8, continuously improving detection accuracy, speed, architectural design, and support for multi-tasking. YOLOv5-seg extends the capabilities of YOLOv5 by introducing a mask head to facilitate instance segmentation. This model consists of three components: the backbone, neck, and head. The backbone extracts numerous features from images, the neck connects the backbone and head to fuse context information, enhancing model robustness, and the head outputs additional mask matrices for instance segmentation, utilizing a box + class + mask approach. In the down-sampling process, YOLOv5 incorporates high-performance complex modules like Conv, C3, and SPFF. The Conv module integrates convolution, BN and SILU activation functions. DS Conv is designed to perform a serpentine convolution operation before the up-sampling process, aiming to fit the target object when the neck fuses context information. The C3 module, a more powerful alternative to ordinary residual blocks, involves a compressed layer of 1 × 1 convolution, a standard 3 × 3 convolution, and an expanded layer of 1 × 1 convolution for residual connections and feature integration. The study enhances the C3 module with DBB. During training, the C3-DBB module is employed, but the first C3 module of the backbone network is retained to extract shallow features. Subsequent convolutional modules use DBB for training, allowing for a rich combination of shallow features and deeper representation capabilities. While maintaining the macro structure of DBB, the microstructure becomes intricate during training. Importantly, DBB is equivalent to a single convolutional layer during inference deployment, aligning with the original inference time cost. the improved YOLOv5-seg model is shown in Fig. 7.

### Model training

This experiment was conducted on a Windows 10 operating system using Python version 3.10.6, PyTorch version 1.13.1, and CUDA 11.1. GPU training and inference were executed on the AutoDL experimental platform, which operates on a Linux platform. The Tesla T4 GPU was utilized for model training, while the Nvidia RTX 4090 was employed for inference purposes. The experiment encompassed a total of 100 epochs, employing a learning rate of 0.01 and a learning rate momentum of 0.937. The weight decay coefficient was set to 0.0005, and the gradient optimization algorithm used was SGD. The default image size was 640 × 640, with a batch size of 32 and 8 threads for processing.

## Results

### Model evaluation

To thoroughly assess the performance of the improved YOLOv5-seg model, this study employs several evaluation metrics, including Precision, Recall, F1-Score, Segment mAP@0.5 (IOU = 0.5), and mAP@0.5:0.05:0.95 with a step size of 0.05. Additionally, the study considers model size and inference speed as crucial indicators for evaluating overall model performance. The Eqs. (19) and (20) for these metrics are outlined below:

19 $$Precision = \frac{TP}{{TP + FP}}$$

20 $$Recall = \frac{TP}{{TP + FN}}$$

**Figure 8:**
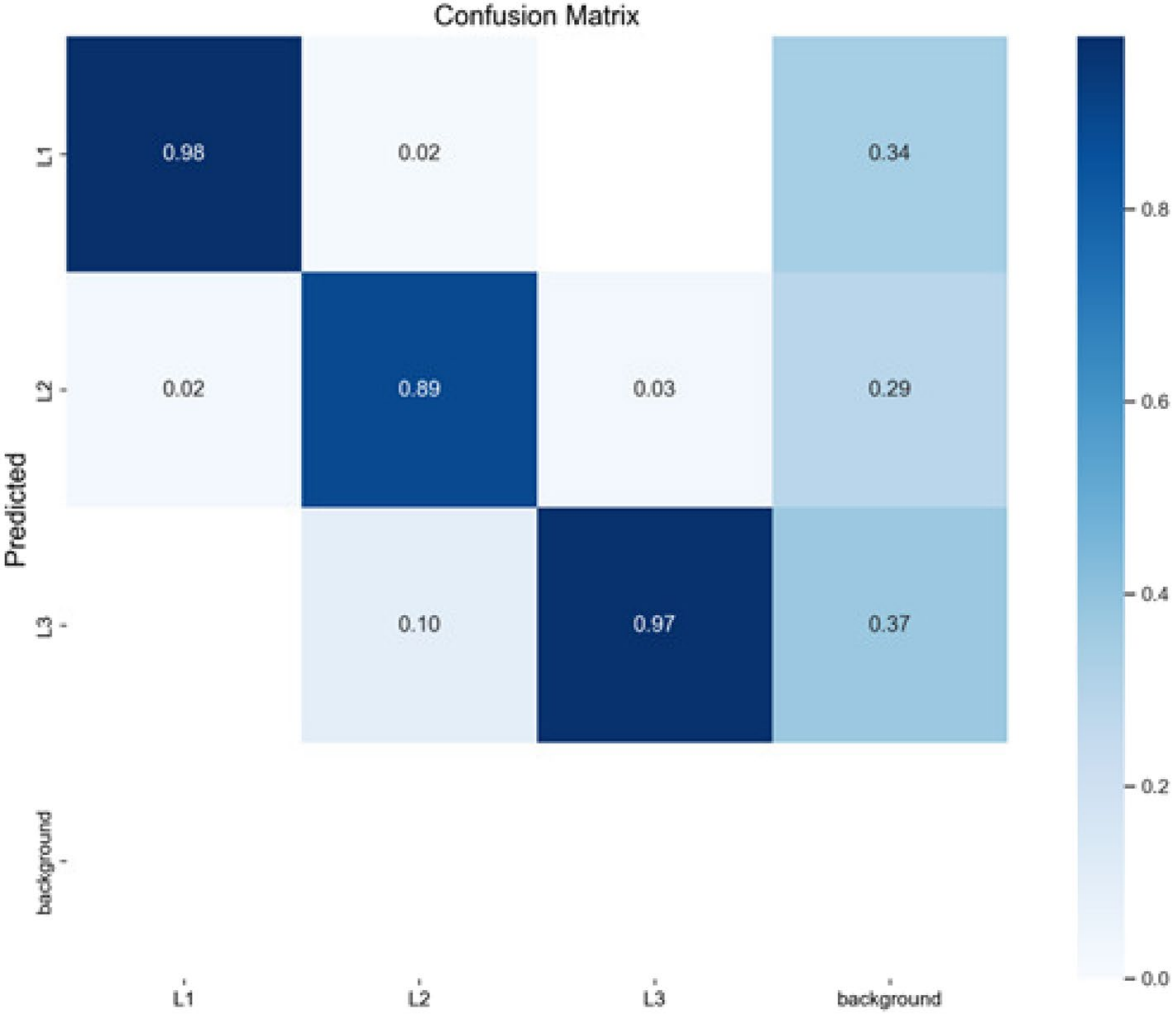
Recognition Confusion Matrix of Bitter Gourd.

Among them, true positive (TP), false positive (FP) and false negative (FN) represent positive samples with correct classification, negative samples with incorrect classification and positive samples with incorrect classification respectively, as shown in Fig. 8. The confusion matrix generated by the improved YOLOv5-seg model in the bitter gourd test set reached an extremely high classification rate of 98% and 97% for L1 and L3 stages of expansion, and the classification accuracy of 89% for L2 stage. In the actual scene, the background interference is strong, and the texture features of the medium and small target bitter gourd are missing in the shooting picture. Some bitter gourds that were taken farther away in the distance images were ignored in the background, while those that were not tagged in the dataset were mostly identified as background. This implies that the model exhibits limitations in recognizing long-range distances and small-scale objects.

F1-score is the harmonic average of precision and recall, and provides a single performance measure through comprehensive consideration. The formula is as follows:

21 $$F1\text{-}score = \frac{2Precision*Recall}{{Precision + Recall}}$$

The Mean Average Precision (mAP) is a metric that quantifies the overall performance of a model by calculating the average precision for each category independently and then averaging these values across all categories. The formula is as follows:

22 $$AP = \mathop \int \limits_0^1 P\left( R \right)d\left( R \right)$$

23 $$mAP = \frac{1}{N}\mathop \sum \limits_1^N A{P_i}$$

**Figure 9:**
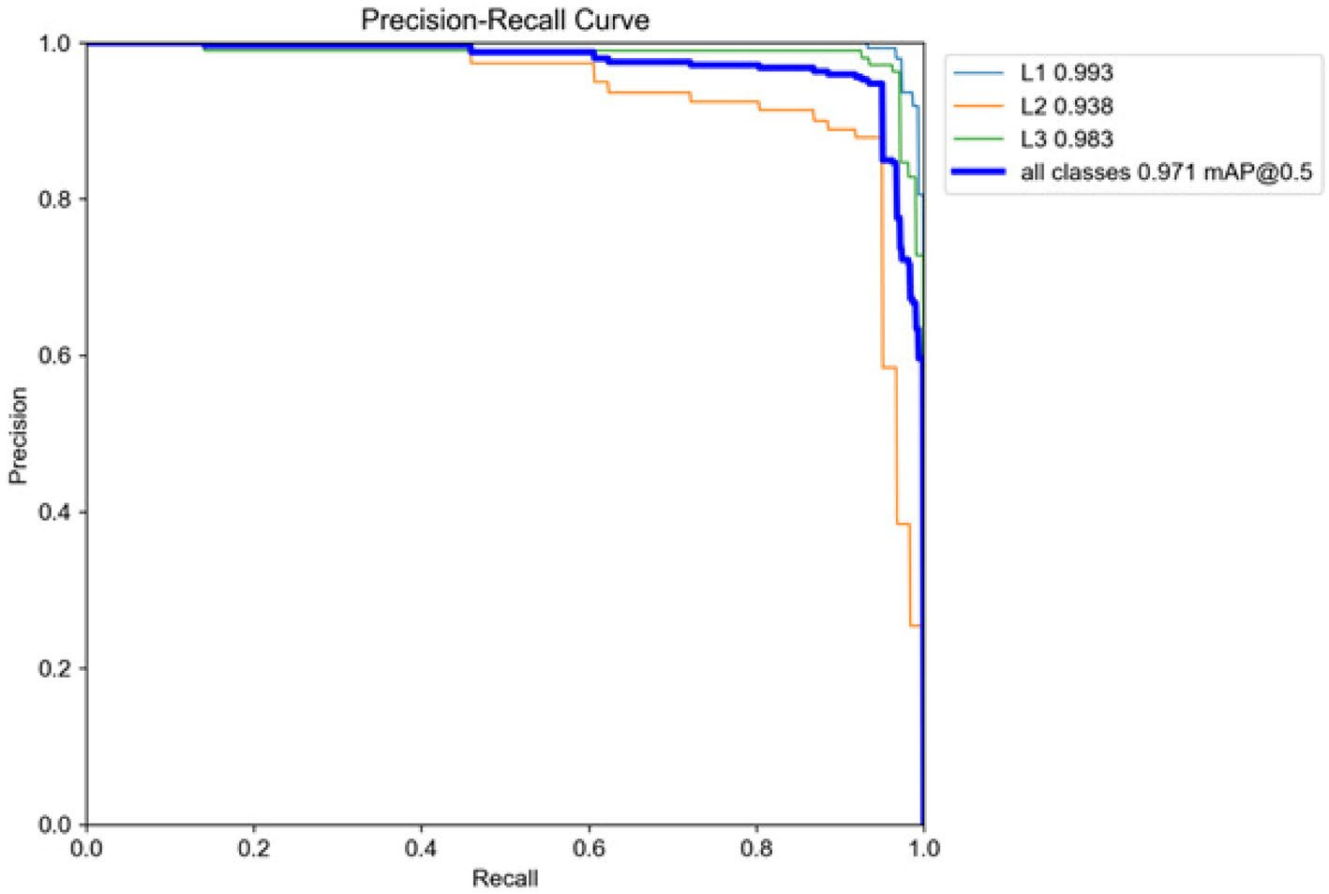
P–R curve.

mAP itself represents the area under the precision-recall curve, offering a measure of the trade-off between precision and recall rates. To ensure that the model must meet the picking conditions in the detection of bitter gourd, which has a high requirement for the precision of the model. This means that the model is more concerned with ensuring that the bitter gourd detected in production actually meets the harvest conditions, rather than paying more attention to the missing cases of bitter gourd that make the model more suitable for different growing environments. Especially in the case of insufficient sample data in L2 stage, this will lead to a decline in the recognition ability of L2 stage. In this way, the model will increase the similarity between the L2 and L1 stages, but the improved model has good performance in both aspects. Figure 9 shows that the improved segmentation model reaches 97.1% in the test set to identify the bitter gourd segment mAP@0.5 during the expansion stage. During the expansion stage of L1 and L3, the identification of bitter gourd will be accurate, basically without errors. However, there may be misclassifications where parts of the L2 stage are mistakenly identified as L1 stage.

### Comparison with other models

To maintain experimental consistency, we ensured uniform training parameter settings across deep learning models. Additionally, we utilized pre-trained model parameters from the COCO dataset to expedite model convergence, thereby achieving optimal performance. The experiment compared the YOLO series detection model with the mainstream case segmentation model. As shown in the following Table 2. the box positioning of target detection was inferior to the case segmentation model in recognizing the maturity of bitter gourd. For background interference and YOLOv5-detect model, it was difficult to accurately identify the surface characteristics of bitter gourd maturity in fine-grained recognition. The mAP@0.5 of the YOLOv5-seg model surpasses that of YOLOv5-detect by 7.3%, with a mere 0.4M increase in model size. YOLOv5-seg goes beyond detecting object boundary boxes. It also produces a pixel-level mask for each object, delivering a more precise contour outline. This enhancement facilitates a deeper understanding of the object's shape and fine details. However, compared with the earlier Mask-RCNN two-stage model, the model size of YOLO series model is smaller and the inference speed is faster. Both the inference speed and the model size exhibit discrepancies of several times. For the YOLACT first-stage instance segmentation model, backbone uses the early Resnet50^40^, and the model size is larger than other models. However, because it is a first-stage model, its reasoning speed reaches 26.55 ms. The mAP@0.5 achieved an impressive 89.5%. The YOLOv5 to YOLOv8 instance segmentation series model has the ability of efficient convolutional block feature extraction and multi-scale feature information fusion, and the model size and reasoning speed are greatly reduced while maintaining high accuracy. On this dataset, Compared with YOLOv8s-seg in segment map@0.5, the YOLOv5s-seg model is 1.2% higher, and the model is smaller and the reasoning speed is 57% faster. This experiment also proves that YOLOv5 series is better and faster than YOLOv8 in some aspects such as model size, inference speed and custom dataset. Finally, based on the improved YOLOv5-seg model, the segment mAP@0.5 can reach 97.1% when the model size and reasoning speed remain unchanged, and the F1-score can reach 94% when the confidence is 0.72.

**Table 2:** Comparison of six models on bitter gourd test set.

Model	Parameters (M)	mAP@0.5 (%)	mAP@0.5:0.95 (%)	F1-score@0.72 (%)	Speed on RTX 4090 (ms)
Mask-RCNN	46.3	87.8	64.4	76.2	58.84
YOLACT-50	33.8	89.5	57.7	74.1	26.55
YOLOv5s-detect	7.2	84.4	69.1	73.4	8.12
YOLOv5n-seg	3.4	75.7	61.3	64.2	8.56
YOLOv5s-seg	7.6	91.7	78.9	88.6	9.02
YOLOv8s-seg	11.8	90.9	75.7	81.8	15.81
Improved YOLOv5s-seg	7.6	97.1	83.5	94.0	8.95

### Ablation experiment

**Figure 10:**
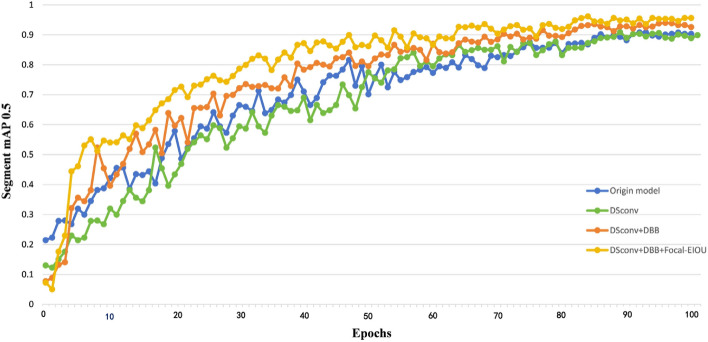
The map of the training process.

**Figure 11:**
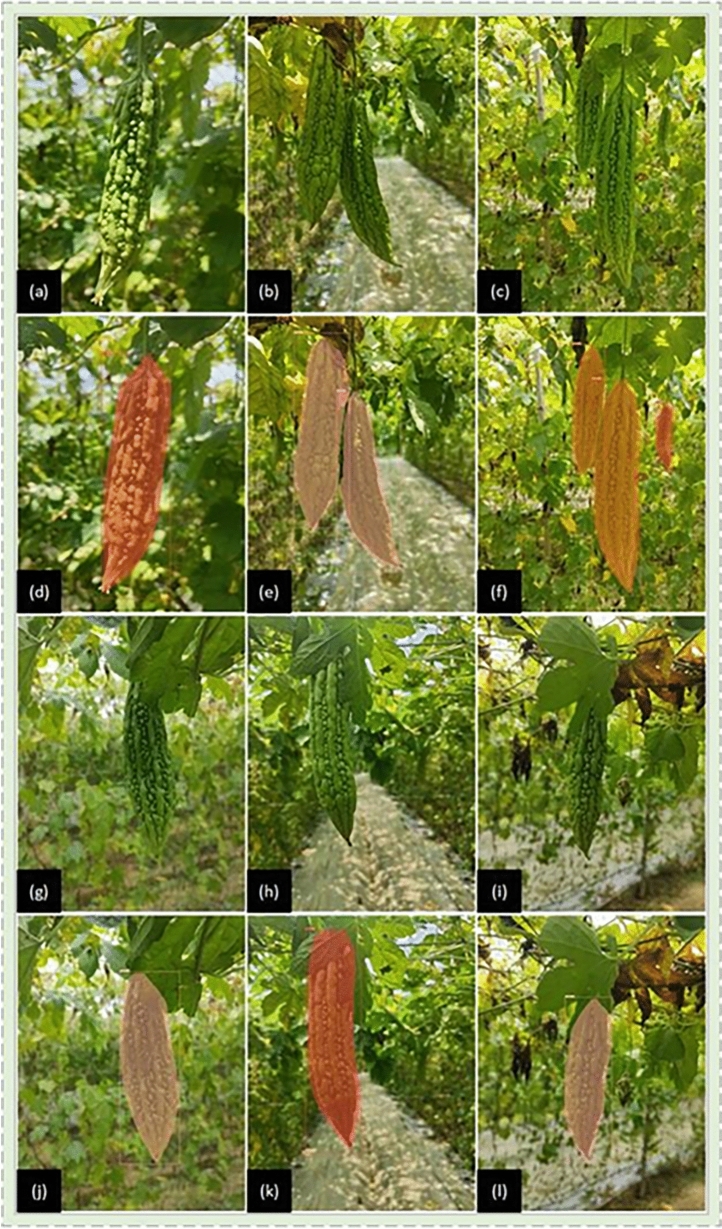
Example of bitter gourd segmentation. (**a**) Bitter Gourd under light change. (**b**,**c**) The fruit partially overlaps. (**g**,**h**,**j**) Small amount of blade obstruction. (**d**) The inference graph of (**a**,**e**,**f**) is the inference graph of (**b**,**c**), (**j**,**k**,**l**) is the inference graph of (**g**,**h**,**j**).

In this experiment, ablation studies were carried out on both the training and validation sets, while the trained model's generalization ability was assessed on the test set. The objective was to evaluate the effectiveness of the improved YOLOv5s-seg model in fine-grained recognition of bitter gourd maturity. Figure 10 illustrates the epoch and segment mAP@0.5 curve, providing a visual representation of the model's performance. Further detailed data is presented in Table 3. There is a marginal increase in model size throughout the training process. In the original YOLOv5s-seg model, due to the loading of pre-training weights, the model performed well at the beginning. Compared with the model introduced with DS Conv, the model added with DS Conv in the training was more fit to the structure of the model, and the training process was more stable. The mAP also has a small increase, but the model size has also increased by 0.83 M offset field learning comes with an overhead. At the same time, by setting reasonable parameters, it is important to keep the continuity of DS Conv in space position and the smoothness of shape change. The imagery obtained through vehicle-mounted systems undergoes dynamic and continual changes. Unlike those captured by mobile phones, these images may offer closer proximity and varied viewing angles, while those from passing vehicles might present greater distances and wider perspectives. Consequently, targets in the images may exhibit diverse sizes and shapes, akin to the varying forms of bitter gourds. DS Conv proves instrumental in several aspects: fitting the target shape more accurately, enhancing spatial information, and adaptively adjusting to target appearances. This adaptability empowers the model to effectively handle targets of varying shapes and sizes, thereby bolstering its generalization capabilities across complex scenarios. In appropriate cases, the introduction of additional offset helps to improve the transformation modeling capability of convolutional neural networks, making them better adapted to a variety of complex input data and task requirements. After the DBB module is merged, the feature extraction capability of the model is strengthened, and the segment map index rises to about 93%. Notably, this enhancement significantly improved the model's ability to detect bitter gourds at the L1 and L3 stages of maturity, with the L1 stage having the most strip-shaped bitter gourds. With dynamic snake convolution focusing on the morphological structure of bitter gourd, DBB enhances the feature extraction ability of the model. Operations at different scales also alleviate the problem of missing long-distance target features to a certain extent. It is acceptable to increase performance at the cost of this additional training resource. Finally, Focal-EIOU loss was introduced. This addition is designed to ease the sample imbalance in the L2 stage and position the rectangular box and mask matrix more accurately. This initially slows down the model's convergence due to its emphasis on challenging samples. However, as training progresses, the model's capability to distinguish between a few categories enhances. In the end, the model achieved an impressive mAP of 95.6%. The performance of the improved YOLOv5-seg is shown in Fig. 11. It is segmented under the condition of illumination variation, leaf occlusion and fruit overlap. The improved YOLOv5-seg model accurately segments conditions such as light variation Fig. 11a, melon overlap Fig. 11b,c, and blade occlusion Fig. 11g,h,j. The segmentation example in Fig. 11k shows that the model can still segment the rough outline of bitter gourd under the condition of blade occlusion, indicating that the model has well learned the shape characteristics of bitter gourd. And can accurately identify and classify.

**Table 3:** The detailed parameter of ablation experiments.

Method	Size	mAP@0.5 (%)	mAP@0.5:0.95 (%)	Training Param (M)	Speed on GPU RTX 4090 (ms)
YOLOv5s-seg	640	90.2	80.9	7.62	8.86
YOLOv5s-seg + DSConv	640	90.6	81.0	8.45	9.12
YOLOv5s-seg + DSConv + DBB	640	92.8	82.9	10.14	8.79
YOLOv5s-seg + DSConv + DBB + Focal-EIOU	640	95.6	83.5	10.14	8.94

## Discussion

Agricultural automation technology is increasingly pivotal in enhancing agricultural production efficiency and addressing challenges such as labor shortages. The accurate estimation of bitter gourd maturity holds significant importance as a crucial component of automated agricultural harvesting. Bitter gourd, being a vital melon, is characterized by a lengthy growth cycle, high yield, a short picking period, and distinct stages of maturity. Automated harvesting of bitter gourd can substantially reduce labor and time costs. Accurate estimation of the Gourd's maturity assists harvesters in making timely decisions, preventing potential quality issues arising from premature or delayed harvesting. The implementation of maturity detection technology not only streamlines processing and production but also enhances supply chain management, providing technical support for intelligent harvesting machines. Timely assessment of fruit maturity in the field further contributes to the improvement of quality and yield.

The automated picking process for bitter gourd relies on a vehicle-mounted system following a predetermined route. Bitter gourd cultivation typically occurs within melon greenhouses, creating favorable conditions for picking equipment between the two ends of fruit development. As depicted in Fig. 2. Additionally, the length and diameter of bitter gourd are crucial factors in estimating their maturity. Under normal growth conditions with sufficient light and nutrient supply, size characteristics serve as important criteria for estimating bitter gourd maturity. The Intel RealSense depth camera provides the necessary interface for this process. When the picking device identifies the target bitter gourd at the appropriate distance, it conducts a detailed assessment. Utilizing generated depth point cloud information, the device obtains bitter gourd size and characteristics, which combined with the maturity discrimination of the improved YOLOv5-seg model, enables accurate judgment of whether bitter gourd meet picking criteria. In actual scenarios, the information on size characteristics also speeds up the judgment of the on vehicle-mounted system. Smaller sizes can be quickly filtered for the next stage of bitter gourd picking.

Fine-grained recognition of the external features of bitter gourd is the key to estimate the maturity of bitter gourd. Box-type positioning detection model has strong background interference and cannot be accurately segmented, while case segmentation can be accurately segmented. Focusing on the features of the object itself, in order to improve the feature extraction ability of the model and reduce the problem of sample imbalance. The improved instance segmentation model based on YOLOv5-seg showed a good performance in estimating the maturity of bitter gourd, but it also brought limitations. The improved model training time is longer, which is less acceptable for large models, and also slows down the model inference on certain GPUs because it cannot be parallelized extensively, unlike conventional convolution computation. Additionally, the recognition performance of bitter gourd at a distance drops linearly, and slightly blurred bitter gourd pictures may be missed or misjudged. At the same time, this study only realized the maturity estimation of Qishen bitter gourd, and some other varieties of bitter gourd have different fine-grained characteristics, such as Indian bitter gourd, the color of its external features will change from light green to dark green, and the gap between the tumor particles will change from elliptical and dense to mountain shaped. The improved YOLOv5-seg model has excellent performance in identifying fine-grained recognition of bitter gourd while focusing on its narrow and long shape. When performing different preprocessing on different types of datasets to highlight the fine-grained characteristics of bitter gourd, the improved YOLOv5-seg can still generalize well to this type because they belong to the same type problem. In future studies, we will generalize this model to other varieties of bitter gourd, and also hope to find a simple and effective method to estimate the maturity of bitter gourd.

## Conclusion

In this study, an improved YOLOv5-seg instance segmentation model is proposed for the estimation of maturity of bitter gourd. In view of the curved and elongated structure of bitter gourd, DS Conv is used to fit the structure of the target object in the neck of the YOLOv5-seg model, focusing on the fine-grained characteristics of bitter gourd. Then, DBB module is introduced to enrich the feature space of convolutional blocks with multi-branch structure, and extract the fine-grained features of the model in a deeper level. The enhanced YOLOv5-seg model improved the recognition ability of bitter gourd segment mAP@0.5 by 2.4% on average in the expansion stage. Finally, Focal-EIOU loss was introduced to solve the sample imbalance in the L2 stage of bitter gourd dataset, accurately positioned the rectangular box and mask, accelerated model convergence and reduced losses. Compared with the original YOLOv5-seg model, the reasoning time of the improved YOLOv5-seg model reaches 8.95 ms. With the reasoning time basically unchanged, segment mAP@0.5 reached 97.1%, an increase of 5.4%. Compared with other instance segmentation models, Mask-RCNN, YOLACT and YOLOv8 improved by 9.3%, 7.6% and 6.2%, respectively, and shortened the reasoning time by 84%, 66% and 43%, respectively. Overall, the improved YOLOv5-seg model has good performance for fine-grained recognition of bitter gourd in the expansion stage, and can detect complex and changeable scenes in real time, but it will bring challenges when identifying smaller targets. When the texture is missing, there are problems of missing detection and misjudgment. In future studies, we are eager to find a simple yet powerful way to estimate the maturity of bitter gourd, or we will continue to optimize deep learning algorithms to improve recognition efficiency.

## Data Availability

The data presented in this study are available on request from the corresponding author.
